# Supplementary material for: The association between arthritis and cognitive function impairment in the older adults: Based on the NHANES 2011–2014
Source: PLoS One. 2024 Sep 27;19(9):e0310546. doi: 10.1371/journal.pone.0310546 (PMC11432873; doi:10.1371/journal.pone.0310546)
Supplement: Attachment — Submitted filename: Response to Reviewers.docx [file pone.0310546.s001.docx]

Reply to Reviewer 1:

Q1:

The author should explain the Abbreviations used in the abstract.

**Reply:**

Thank you for underlining this deficiency. We have modified our text as advised.

Changes in the text:

This section was modified according to the comment (Lines 26-27, page 2).

Q2:

The author should explain the Abbreviations used in the table below for better and faster interpretation of the data.

**Reply:**

Thank you for underlining this deficiency. We have explained the full meaning of the Abbreviations at the bottom of the chart.

Changes in the text:

This section was modified according to the comment (Lines 338-348, page 17).

Reply to Reviewer 2:

Major:

Q1:

The primary concern is about the methodology utilized in the current study to conclude the correlation. To the best of this reviewer’s understanding, the Consortium to Establish a Registry for Alzheimer’s disease (CERAD) should be including tests that measure word-related learning (CERAD-IR) and memory components (CERAD-DR). Reviewer would like to see more specific details for other parameters like Word List Learning Test (WLLT), the Word List Recall Test (WLRT), and Intrusion Word Count Test (WLLT-IC and WLRT-IC).

**Reply:**

Thank you for underlining this deficiency. We re-analyzed the data statistically as you suggested, changing to four cognitive testing methods (Immediate Recall test (IRT), Delayed Recall test (DRT), Animal Fluency test (AFT), and Digit Symbol Substitution test (DSST)) for analysis. But unfortunately, the Intrusion Word Count Test has a skewed distribution of frequencies and further regression analysis cannot be performed. The following chart shows the frequency distribution of the Intrusion Word Count Test

Changes in the Tables and Figures:

The corresponding data in Table 1-3 and Figure 2-3 have been updated.

Q2:

Reviewer have concerns about the analysis and presentation of results in particular. Overall, the way that the many statistical analyses were presented are somewhat confusing, and I believe this data need to be present in a clear format for a general reader. There need to have a graphical presentation of the major findings about Logistic relationship between cognitive function and arthritis after the adjustment for sociodemographic factors.

**Reply:**

Thank you for underlining this deficiency. We have re-presented the data from the three models of the original Table 4 multivariate logistic regression as forest plots based on your suggestion.

Changes in the Figures:

Figure 3.

Q3:

How about depression and including Patient Health Questionnaire-9 (PHQ-9) in your data mining and correlates that with Arthritis?

**Reply:**

Thank you for your advice. In response to your suggestion we investigated whether there is a correlation between arthritis and depression (PHQ-9 score). Also, for socio-demographic, behavioral and health factors we built three models for logistic regression analysis and the following figure shows the visualization of the multivariate logistic regression for arthritis and depression.

Q4:

Results section need to rewrite, with section headings with clear interpretation of particular findings.

**Reply:**

Thank you for underlining this deficiency. We have made changes in the results section as suggested.

Changes in the text:

This section was modified according to the comment (Lines 186-198, pages 9-10; Lines 203-212, pages 10-11).

Minor:

Q1:

In Methods: Please expand your Statistical analysis approach in detail.

**Reply:**

Thank you for underlining this deficiency. We have elaborated the statistical analysis methods used in this study as suggested.

Changes in the text:

This section was modified according to the comment (Lines 144-168, pages 7-8).

Q2:

Don’t use the words that may have negative connotations, such as “the aged,” “elderly,” “senior,” “senior citizen,” rather use more polite words “older adults,” “older populations,” Please change it throughout the manuscript.

**Reply:**

Thank you for underlining this deficiency. We apologize for any inconvenience caused by our inappropriate expression. We have modified our inappropriate language as suggested.

Changes in the text:

This section was modified according to the comment (Line 147, page 7; Line 171, page 9; Line 207, 211, page 10; Line 211, 216, page 11; Lines 274-275, page 14).

Q3:

Discussion need to be expand and please propose the mechanism in depth about the correlation of arthritis and cognitive decline by citing recent literature.

**Reply:**

Thank you for underlining this deficiency. In response to your suggestion, we have added a discussion of the mechanisms by which arthritis is associated with cognitive decline in Section 4, focusing on the impact on cognitive function of the four main aspects of arthritis-induced chronic inflammation, cardiovascular risk, medication side effects, and reduced quality of life.

Changes in the text:

This section was modified according to the comment (Lines 227-232, pages 11-12; Lines 283-296, pages 14-15).

Q4:

Study published in 2021 on the same subject that describe that No association between rheumatoid arthritis and cognitive impairment in a cross-sectional national sample of older U.S. adults. PMID: 34404491. Please discuss this in your discussion.

**Reply:**

Thank you for underlining this deficiency. We have added this part of the discussion

Changes in the text:

This section was modified according to the comment (Lines 248-260, pages 12-13).

Q5:

Explain how this data and conclusions from your study could be useful in clinic.

**Reply:**

Thank you for underlining this deficiency. We've added this part to the text.

Changes in the text:

This section was modified according to the comment (Lines 296-301, page 15).

Q6:

Describe the limitations of your study and data mining in the discussion section.

**Reply:**

Thank you for underlining this deficiency. We've added this part to the text.

Changes in the text:

This section was modified according to the comment (Lines 302-320, pages 15-16).
