# Supplementary material for: The association between arthritis and cognitive function impairment in the older adults: Based on the NHANES 2011–2014
Source: PLoS One. 2024 Sep 27;19(9):e0310546. doi: 10.1371/journal.pone.0310546 (PMC11432873; doi:10.1371/journal.pone.0310546)
Supplement: Attachment — Submitted filename: Response to Reviewers.docx [file pone.0310546.s002.docx]

Reply to Reviewer 1:

Change elderly to "older adults" in these sentences:

1) In your title, "Line 2 Impairment in the Elderly: Based on the NHANES 2011-2014"

2) Abstract, line 37, elders.

3) Line 86 impairment incidence among the elderly in the United States.

4) Line 300 study not only for the personal quality of life of elderly people with arthritis

**Reply:**

Thank you for underlining this deficiency. We apologize for any inconvenience caused by our inappropriate expression.

Changes in the text:

1) In your title, "Line 2 Impairment in the Elderly: Based on the NHANES 2011-2014"

This phrase was modified according to the comment (Line 2, page 1).

2) Abstract, line 37, elders.

This phrase was modified according to the comment (Line 37, page 2).

3) Line 86 impairment incidence among the elderly in the United States.

This phrase was modified according to the comment (Line 86, page 5).

4) Line 300 study not only for the personal quality of life of elderly people with arthritis

This phrase was modified according to the comment (Line 300, page 15).

Reply to Reviewer 2:

minor changes/suggestions

line 1 title between instead of among

line 28 were instead of was

line 29 patients instead of subjects

line 44 were instead of are

line 269 Alzheimer disease abbreviation repeated (AD)

line 319 patients instead of subjects

**Reply:**

Thank you for underlining this deficiency. We apologize for any inconvenience caused by our inappropriate expression.

Changes in the text:

1) line 1 title between instead of among

This phrase was modified according to the comment (Line 1, page 1).

2) line 28 were instead of was

This phrase was modified according to the comment (Line 28, page 2).

3) line 29 patients instead of subjects

This phrase was modified according to the comment (Line 29, page 2).

4) line 44 were instead of are

This phrase was modified according to the comment (Line 44, page 2).

5) line 269 Alzheimer disease abbreviation repeated (AD)

This phrase was modified according to the comment (Line 268, page 13).

6) line 319 patients instead of subjects

This phrase was modified according to the comment (Line 319, page 16).
